# Antitherapeutic antibody-mediated hepatotoxicity of recombinant human Apo2L/TRAIL in the cynomolgus monkey

**DOI:** 10.1038/cddis.2016.241

**Published:** 2016-08-11

**Authors:** Christina L Zuch de Zafra, Avi Ashkenazi, Walter C Darbonne, Melissa Cheu, Klara Totpal, Shirley Ortega, Heather Flores, Mark D Walker, Bruce Kabakoff, Bert L Lum, Barbara J Mounho-Zamora, Scot A Marsters, Noël O Dybdal

**Affiliations:** 1 Safety Assessment, Genentech, Inc., 1 DNA Way, South San Francisco, CA 94080, USA; 2 Department of Cancer Immunology, Genentech, Inc., 1 DNA Way, South San Francisco, CA 94080, USA; 3 Oncology Biomarker Development, Genentech, Inc., 1 DNA Way, South San Francisco, CA 94080, USA; 4 BioAnalytical Sciences, Genentech, Inc., 1 DNA Way, South San Francisco, CA 94080, USA; 5 Late Stage Pharmaceutical Development, Genentech, Inc., 1 DNA Way, South San Francisco, CA 94080, USA; 6 Charles River Laboratories Inc. (formerly Sierra Biomedical), 587 Dunn Circle, Sparks, NV 89431, USA; 7 Early Stage Pharmaceutical Development, Genentech, Inc., 1 DNA Way, South San Francisco, CA 94080, USA; 8 Clinical Pharmacology, Genentech, Inc., 1 DNA Way, South San Francisco, CA 94080, USA; 9 Toxicology Department, Amgen Inc., One Amgen Center Drive, Thousand Oaks, CA 91320, USA

## Abstract

Apo2L/TRAIL is a member of the tumor necrosis factor superfamily and an important inducer of apoptosis. Recombinant human (rhu) Apo2L/TRAIL has been attractive as a potential cancer therapeutic because many types of tumor cells are sensitive to its apoptosis-inducing effects. Nonclinical toxicology studies were conducted to evaluate the safety of rhuApo2L/TRAIL for possible use in humans. The cynomolgus monkey was chosen for this safety assessment based on high protein sequence homology between human and cynomolgus Apo2L/TRAIL and comparable expression of their receptors. Although hepatotoxicity was observed in repeat-dose monkey studies with rhuApo2L/TRAIL, all animals that displayed hepatotoxicity had developed antitherapeutic antibodies (ATAs). The cynomolgus ATAs augmented the cytotoxicity of rhuApo2L/TRAIL but not of its cynomolgus counterpart. Of note, human and cynomolgus Apo2L/TRAIL differ by four amino acids, three of which are surface-exposed. *In vivo* studies comparing human and cynomolgus Apo2L/TRAIL supported the conclusion that these distinct amino acids served as epitopes for cross-species ATAs, capable of crosslinking rhuApo2L/TRAIL and thus triggering hepatocyte apoptosis. We describe a hapten-independent mechanism of immune-mediated, drug-related hepatotoxicity – in this case – associated with the administration of a human recombinant protein in monkeys. The elucidation of this mechanism enabled successful transition of rhuApo2L/TRAIL into human clinical trials.

Apo2L/TRAIL, a member of the tumor necrosis factor (TNF) ligand superfamily, is critically involved in the process of apoptosis.^1, 2^ Apo2L/TRAIL is a type II transmembrane protein with an extracellular portion that can bind to death receptors DR4 (TRAIL-R1) and DR5 (TRAIL-R2), and decoy receptors DcR1 (TRAIL-R3), DcR2 (TRAIL-R4) and osteoprotegerin (OPG). Apo2L/TRAIL binding to DR4 and/or DR5^3, 4^ initiates an intracellular signal cascade culminating in apoptosis.^5^ Apo2L/TRAIL pathway activation is efficacious in preclinical cancer models.^6, 7, 8, 9, 10, 11^ Unlike most normal cells, many tumor cell types are sensitive to Apo2L/TRAIL,^12, 13, 14, 15, 16, 17^ making the pathway attractive to anticancer therapeutic development. However, clinical studies to date have not shown broad efficacy of Apo2L/TRAIL-related agents,^18^ generating interest in the development of more potent agonists targeting this pathway.^19, 20, 21^ Understanding the safety profile of agents that target the Apo2L/TRAIL receptors is critical for the development of second-generation molecules that may prove to be more efficacious.

Hepatotoxicity has been a major limitation for the clinical use of TNF superfamily members.^22, 23, 24^ A small subset of normal cell types that express DR4 and DR5, including hepatocytes, has been shown to be susceptible to Apo2L/TRAIL-induced injury^23^ and administration of an anti-Fas antibody to mice resulted in hepatotoxicity and mortality.^24, 25^ However, hepatotoxicity has been shown to be the result of physicochemical attributes, namely the propensity to self-aggregate with the resulting downstream signaling, of the recombinant ligand used (i.e., polyhistidine-tagged or leucine-zipper Apo2L/TRAIL).^26^

Recombinant human (rhu) Apo2L/TRAIL (dulanermin; developed through collaboration between Genentech, South San Francisco, CA, USA and Amgen, Thousand Oaks, CA, USA) is comprised of a portion of the extracellular domain of the natural ligand (amino acids 114–281) that can mimic its activity. RhuApo2L/TRAIL is a stable trimeric form of Apo2L/TRAIL, which has been shown to be non-hepatotoxic in *in vitro* studies.^26^

RhuApo2L/TRAIL was evaluated in nonclinical toxicology studies to enable its use as a cancer therapy. Based on sequence similarities between human and cynomolgus monkey Apo2L/TRAIL or Apo2L/TRAIL receptors (84–99% identity^3^), and the comparable expression and tissue distribution of DR4, DR5 and OPG in the two primates, the cynomolgus monkey was the most relevant species for these studies. The clinical development plan included treatment with rhuApo2L/TRAIL alone and in combination with nephrotoxic chemotherapeutic agents including cisplatin. Thus, renal injury model studies were conducted using cisplatin to assess the potential of rhuApo2L/TRAIL to exacerbate clinically relevant renal injury. Despite the use of stable, trimeric rhuApo2L/TRAIL, hepatotoxicity was observed. The unexpected results engendered the hypothesis that cynomolgus anti-rhuApo2L/TRAIL antibodies promoted ligand crosslinking and subsequent aggregation of death receptors, thereby leading to apoptosis of cynomolgus hepatocytes. This was subsequently tested by analysis of cynomolgus serum as well as functional studies. The findings reported here describe a unique mechanism of immune-mediated hepatotoxicity, involving the development of species-specific crosslinking antibodies that act together with their target to elicit a potent biological effect on hepatocytes.

## Results

### RhuApo2L/TRAIL toxicology studies demonstrate hepatotoxicity in cynomolgus monkeys

**Figure 1:**
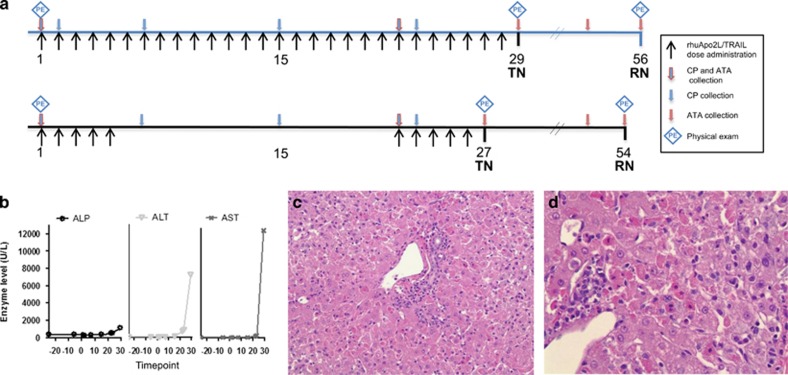
Evidence of hepatotoxicity in 4-week repeat-dose toxicology study of rhuApo2L/TRAIL. (**a**) Study design schema. Black arrows indicate days of rhuApo2L/TRAIL administration (top line: 0, 10, 30 or 100 mg/kg rhuApo2/TRAIL (*n*=4–6/sex/group) daily for 28 days; bottom line: 100 mg/kg rhuApo2/TRAIL on days 1–5 and 22–26 (*n*=6/sex)); blue arrows outlined in red indicate timing of blood sampling for clinical pathology (CP) and ATA/crosslinking activity analyses; blue arrows indicate timing of blood sampling for CP analysis; red arrows indicate timing of blood sampling for ATA/crosslinking activity analyses. Blue diamonds indicate timing of physical examinations. TN, terminal necropsy; RN, recovery necropsy. (**b**) Alterations in liver enzyme levels in one high-dose (100 mg/kg) group animal administered rhuApo2L/TRAIL daily for 28 days. ALP, alkaline phosphatase; ALT, alanine aminotransferase; AST, aspartate aminotransferase. (**c**) Representative hematoxylin and eosin-stained liver sections at × 20 magnification. (**d**) Representative hematoxylin and eosin (H&E)-stained liver sections at × 40 magnification. H&E-stained sections depict rhuApo2L/TRAIL-associated hepatic pathology from the same high-dose (100 mg/kg) group animal as in panel (**b**). Disrupted hepatic architecture was observed, lymphocytic infiltrates were evident and many vacuolated and necrotic hepatocytes were present

Evidence of hepatotoxicity accumulated over the course of the toxicology program. In the 4-week repeat-dose study (Figure 1a), elevated liver enzyme levels (serum alkaline phosphatase (ALP) (1.4 × upper limit of normal range (ULN)), alanine aminotransferase (ALT) (57 × ULN) and aspartate aminotransferase (AST) (134 × ULN)) were noted in a single high-dose (100 mg/kg; daily administration) animal at the end of the dosing period (days 23 and 29; Figure 1b). Consistent with the enzyme abnormalities, the liver was mottled correlating with severe acute hepatocellular necrosis associated with subacute inflammation evident microscopically (Figures 1c and d). Clinical pathology values for all other animals (*n*=51) remained within the normal range and there were no other anatomic observations of hepatotoxicity.

**Figure 2:**
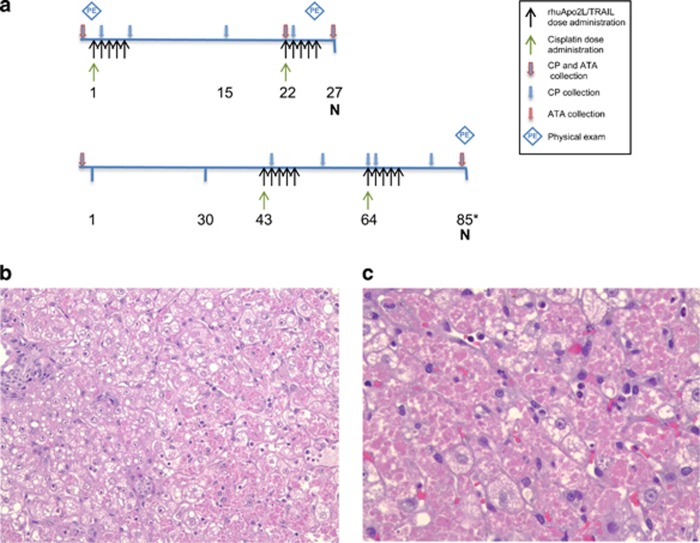
Evidence of hepatotoxicity in two renal injury model studies using concomitant administration of cisplatin and rhuApo2L/TRAIL. (**a**) Study design schemas. Black arrows indicate rhuApo2L/TRAIL administration (top line: 0, 30 or 100 mg/kg rhuApo2/TRAIL (*n*=2/sex/group) on days 1–5 and 22–26; bottom line: 100 mg/kg rhuApo2/TRAIL on days 43–47 and 64–68 (*n*=6/sex)); green arrows indicate cisplatin administration (4 mg/kg; top line: days 1 and 22, bottom line: days 43 and 64); blue arrows outlined in red indicate timing of blood sampling for clinical pathology (CP) and ATA/crosslinking activity analyses; blue arrows indicate timing of blood sampling for CP analysis; red arrows indicate timing of blood sampling for ATA/crosslinking activity analyses. Blue diamonds indicate timing of physical examinations. N, necropsy (*day 85 or 86). (**b**) Representative hematoxylin and eosin (H&E)-stained liver section at × 20 magnification, and (c) representative H&E-stained section at × 40 magnification, depicting rhuApo2L/TRAIL-associated hepatic pathology in an animal in the concomitant cisplatin (4 mg/kg)+rhuApo2L/TRAIL (100 mg/kg) group. Disrupted hepatic architecture was observed and many vacuolated and necrotic hepatocytes were present

**Figure 3:**
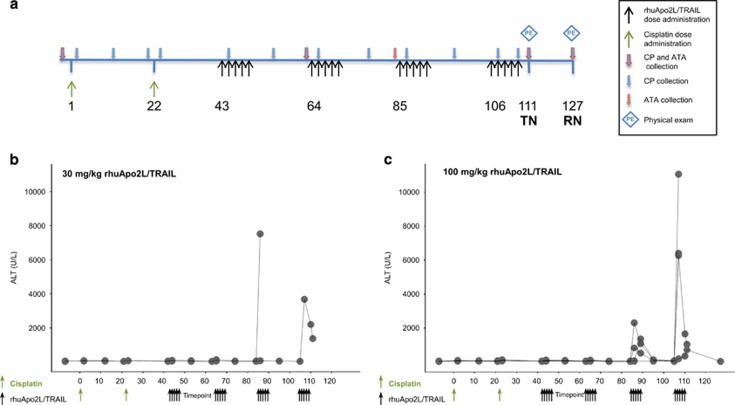
Evidence of hepatotoxicity in renal injury model study using sequential administration of cisplatin and rhuApo2L/TRAIL. (**a**) Study design schema. Black arrows indicate rhuApo2L/TRAIL administration (0, 10, 30 or 100 mg/kg rhuApo2/TRAIL (*n*=4–6/sex/group)) on days 43–47, 64–68, 85–89 and 106–110; green arrows indicate cisplatin administration (4 mg/kg) on days 1 and 22; blue arrows outlined in red indicate timing of blood sampling for clinical pathology (CP) and ATA/crosslinking activity analyses; blue arrows indicate timing of blood sampling for CP analysis; red arrows indicate timing of blood sampling for ATA/crosslinking activity analyses. Blue diamonds indicate timing of physical examinations. TN, terminal necropsy; RN, recovery necropsy. (**b** and **c**) Elevations in ALT levels in individual animals following sequential treatment with cisplatin (4 mg/kg) and 30 mg/kg rhuApo2L/TRAIL or 100 mg/kg rhuApo2L/TRAIL. Dose administration time points are indicated by green arrows (cisplatin) and black arrows (rhuApo2L/TRAIL)

Clinical and histopathological (Figures 2b and c) observations of liver injury were noted following concomitant administration of cisplatin+rhuApo2L/TRAIL (Figure 2a). However, the evidence of rhuApo2L/TRAIL-induced hepatotoxicity was strongest following sequential administration of cisplatin+rhuApo2L/TRAIL (Figure 3a). Transient increases in serum ALT levels were observed in individual animals in all rhuApo2L/TRAIL dose groups (Table 1). The magnitude of the changes increased with consecutive dosing cycles (Figures 3b and c).

In the study using sequential administration of cisplatin and rhuApo2L/TRAIL, the number of cycles of intermittent (5d q3w) treatment was increased from 2 (in the 4-week repeat-dose study and the studies utilizing concomitant cisplatin+rhuApo2L/TRAIL administration) to 4. The increase in treatment duration was associated with an increase in the incidence and the severity of hepatotoxicity. Although the sequential administration of cisplatin and the 10 mg/kg dose of rhuApo2L/TRAIL was well tolerated, treatment-related toxicities at higher doses of rhuApo2L/TRAIL resulted in several deaths or unscheduled euthanasias. Two animals in the 30 mg/kg rhuApo2L/TRAIL group (days 87 and 111), and one animal in the 100 mg/kg group were killed early (day 110). Additionally, two animals died (one on day 30, before receiving rhuApo2L/TRAIL, and one on day 108). Among the animals that died or were euthanized early, low food consumption, decreased activity, hunched appearance, emesis and/or icterus were observed several days preceding death. Among the animals that survived to scheduled euthanasia, treatment-related clinical observations included decreased activity, decreased appetite, decreased skin turgor and hunched appearance. In four of five animals (two from the 30 mg/kg and three from the 100 mg/kg rhuApo2L/TRAIL groups) euthanized early or that died during the study, paleness, discoloration/mottling and friability of the liver were noted macroscopically and correlated with microscopic observations of marked hepatocellular necrosis and/or vacuolation (30 mg/kg group animals), or mild hepatocellular necrosis and moderate to marked hepatocellular vacuolation (100 mg/kg group animals). Among animals euthanized at scheduled time points, mild, diffuse discoloration of the liver was noted in one animal in both the 30 and 100 mg/kg groups. Minimal to mild hepatic necrosis, minimal to moderate hepatocyte vacuolation and/or hepatocyte mitotic figures were observed microscopically in one animal in the 30 mg/kg group and two animals in the 100 mg/kg group, respectively.

### Anti-rhuApo2/TRAIL antibodies correlate with hepatotoxicity

Antitherapeutic antibodies (ATAs) were assessed in all studies, initially by enzyme-linked immunosorbent assay (ELISA) but later (renal injury model and investigative studies) also by radioimmunoprecipitation (RIP) assay which had greater drug tolerance and sensitivity. By ELISA, no anti-rhuApo2L/TRAIL antibodies were detected in animals from either the 4-week study or the first renal injury model study (concomitant cisplatin+rhuApo2L/TRAIL; Figure 2a, top). In contrast, 3 of 12 animals from the concomitant cisplatin+100 mg/kg rhuApo2L/TRAIL group in the second renal injury model study (Figure 2a, bottom) had ATAs that were detectable by ELISA (Table 1). The difference in ATA responses in concomitant treatment groups between studies was likely due to the timing of the antibody measurements and the inclusion of a treatment-free recovery period in the second renal injury model study.

In the study using sequential administration of cisplatin and rhuApo2L/TRAIL, both ELISA and RIP assays were employed to measure ATAs. By ELISA, ATAs were detected in 1 of 8 animals in the 10 mg/kg rhuApo2L/TRAIL group, 1 of 8 animals in the 30 mg/kg rhuApo2L/TRAIL group and 5 of 12 animals in the 100 mg/kg rhuApo2L/TRAIL group (Table 1). Antibody titers were comparable across dose levels. The timing of ATA development inversely correlated with the dose level, with ATAs evident after three cycles of treatment in the 10 mg/kg group and after two cycles of treatment in the 30 and 100 mg/kg groups. The RIP assay detected ATAs at earlier time points in animals that were ATA-positive by ELISA. Additionally, the RIP assay detected ATAs in several animals that were ATA-negative by ELISA (five additional ATA-positive animals in each of the 30 and 100 mg/kg groups), confirming the greater sensitivity of the RIP method. Two animals in the 100 mg/kg group that died before the end of the study were RIP antibody positive on day 63 (one day before the start of cycle 2). No antibodies were detected in the third animal that died during the study; however, only the ELISA was used to assay samples from this animal due to the lack of sufficient samples to perform the RIP assay (Table 1). Except for this animal, clinical and/or microscopic evidence of hepatotoxicity was observed only in ATA-positive animals. Hepatotoxicity was not observed in animals that were ATA-negative.

To test the hypothesis that ATAs may have enhanced the agonistic activity of rhuApo2L/TRAIL toward hepatocytes, the biological activity of the ATAs from a subset of antibody-positive animals was analyzed in the Jurkat cytotoxicity assay. The Jurkat T leukemia cell line is resistant to trimeric rhuApo2L/TRAIL but becomes sensitive upon crosslinking of the ligand when marked by a FLAG epitope tag and crosslinked with anti-FLAG antibodies.^26^ Serum samples were incubated with human Jurkat cells in the presence of trimeric rhuApo2L/TRAIL or rcynoApo2L/TRAIL. Decreased cell viability indicated the presence of ATAs that could crosslink rhuApo2L/TRAIL with subsequent aggregation of death receptors, resulting in apoptosis.

Serum from 8 of 12 animals concomitantly administered cisplatin+100 mg/kg rhuApo2L/TRAIL was assayed. ATAs capable of crosslinking rhuApo2L/TRAIL were detected in 6 of 8 animals (Table 1). The specificity of the crosslinking activity was assessed by incubating the serum samples with rcynoApo2L/TRAIL; Jurkat cytotoxicity was only observed in one animal that had a very high ATA titer, suggesting some minimal nonspecific crossreactivity with rcynoApo2L/TRAIL (data not shown).

Serum from animals sequentially administered cisplatin+rhuApo2L/TRAIL was also tested in the Jurkat cytotoxicity assay. Again, decreased Jurkat cell viability was noted after incubation with rhuApo2L/TRAIL (1 of 8 animals in the 10 mg/kg group, 2 of 8 animals in the 30 mg/kg group and 5 of 12 animals in the 100 mg/kg group) (Table 1). No cytotoxicity was observed following incubation of these samples with rcynoApo2L/TRAIL, suggesting specificity of the ATAs against rhuApo2L/TRAIL. Samples from two vehicle-treated animals and 2 of 3 animals in the 30 mg/kg group were negative in the Jurkat cytotoxicity assay, and the RIP assay indicated a corresponding lack of ATAs.

### Species-specific ATAs mediate Apo2L/TRAIL-induced hepatotoxicity

**Figure 4:**
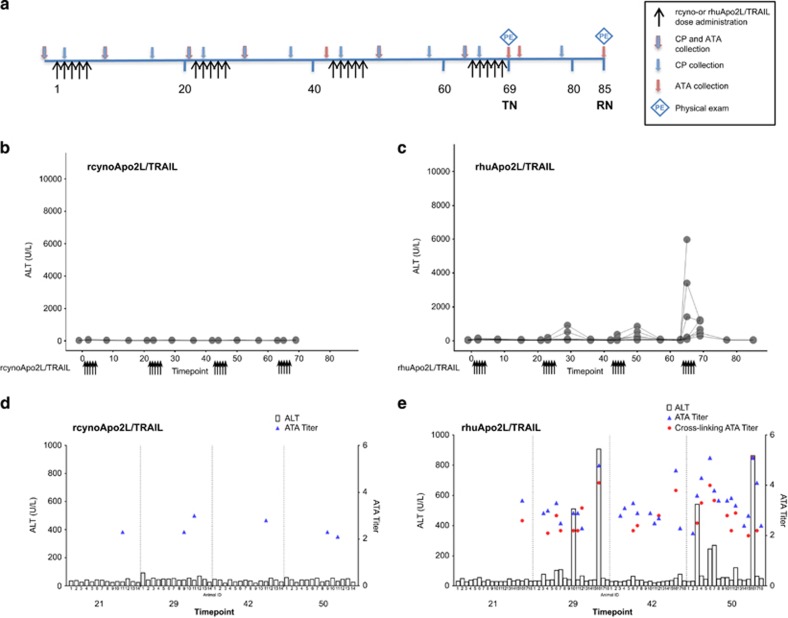
Evidence of hepatotoxicity in investigative study comparing recombinant cynomolgus monkey and human Apo2L/TRAIL. (**a**) Study design schema. Black arrows indicate days of rhuApo2L/TRAIL or rcynoApo2L/TRAIL administration (100 mg/kg; days 1–5, 22–26, 43–47 and 64–68 (*n*=4–9/sex/group)); blue arrows outlined in red indicate timing of blood sampling for clinical pathology (CP) and ATA/crosslinking activity analyses; blue arrows indicate timing of blood sampling for CP analysis; red arrows indicate timing of blood sampling for ATA/crosslinking activity analyses. Blue diamonds indicate timing of physical examinations. TN, terminal necropsy; RN, recovery necropsy. (**b**) ALT elevations following treatment with 100 mg/kg rcynoApo2L/TRAIL. (**c**) ALT elevations following treatment with 100 mg/kg rhuApo2L/TRAIL. (**d**) Individual animal data showing the correspondence between ALT (bars), ATAs (blue triangles) and crosslinking ATAs (red circles) on study days 21, 29, 42 and 50 following treatment with 100 mg/kg rcynoApo2L/TRAIL. (**e**) Individual animal data showing the correspondence between ALT (bars), ATAs (blue triangles) and crosslinking ATAs (red circles) on study days 21, 29, 42 and 50 following treatment with 100 mg/kg rhuApo2L/TRAIL

**Figure 5:**
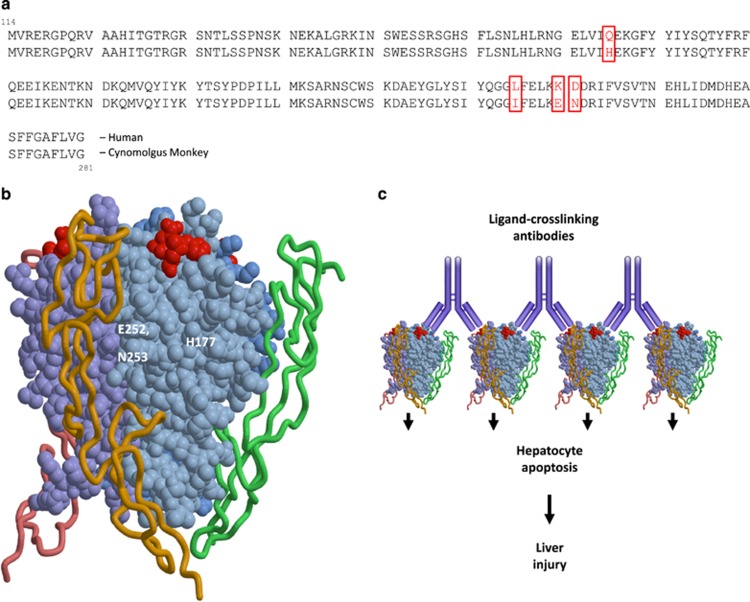
(**a**) Sequence comparison of human and cynomolgus monkey Apo2L/TRAIL, residues 114–281 of the extracellular domain, showing the positions of the distinct amino acids between the two species. (**b**) Three-dimensional rendering of human Apo2L/TRAIL interacting with a membrane-bound death receptor. Shown in red are three of the four amino acids (H177, E252 and N253) that differ between the human and cynomolgus monkey proteins and are distal to the receptor binding region of the Apo2L/TRAIL protein. (**c**) A rendering of the mechanism of toxicity, wherein anti-Apo2L/TRAIL antibodies bind to Apo2L/TRAIL, bringing them into close proximity with one another and thereby clustering death receptors at the surface of the hepatocyte. Such receptor clustering can lead to excessive intracellular apoptosis signaling and consequent hepatotoxicity

The human and cynomolgus monkey Apo2L/TRAIL protein sequences differ by only four amino acids (Figure 5a); however, three of these are in exposed regions of the protein and could serve as an immunogenic epitope(s) for the development of monkey anti-human Apo2L/TRAIL antibodies (Figure 5b). The observation of hepatotoxicity only in animals that developed ATAs (Table 1) supported the hypothesis that anti-rhuApo2L/TRAIL antibodies were capable of crosslinking rhuApo2L/TRAIL and inducing hepatotoxicity. This hypothesis was tested in a study of cynomolgus monkey Apo2L/TRAIL (rcynoApo2L/TRAIL) *versus* rhuApo2L/TRAIL (Figure 4a). Administration of 100 mg/kg rcynoApo2L/TRAIL was well tolerated, with no observed increase in ALT (Figure 4b) or effect on clinical pathology, or macro- or microscopic pathology (data not shown).

In contrast, increased ALT (Figure 4c), often accompanied by increases in AST, LDH, AP and/or bilirubin levels (data not shown), was observed in 5 of 18 animals in the rhuApo2L/TRAIL group. These increases occurred as early as cycle 2 (two animals), and generally increased in magnitude with successive cycles of treatment. The values of these analytes returned to baseline levels between treatment cycles. One animal in the rhuApo2L/TRAIL group died on day 65 (following the second dose in cycle 4); emesis, hypothermia, hypoactivity, pallor and maxillofacial edema were observed on day 65 before death. All other animals in this group survived to scheduled euthanasia, and no other clinical observations were noted.

In the rhuApo2L/TRAIL-treated animal that died prior to the end of the study, the liver was discolored and friable correlating with the microscopic observation of diffuse, severe hepatocellular necrosis. These changes correlated with marked ALT elevations, indicative of severe hepatotoxicity (Figure 4c and Table 2). Macroscopic changes were observed in only one other animal in the rhuApo2L/TRAIL group at terminal necropsy, and consisted of diffuse pale discoloration of the liver correlating with the microscopic findings of moderate diffuse microvesicular lipidosis of hepatocytes, accompanied by a mild increase in hepatocellular mitotic activity and mild infiltration of neutrophils in the sinusoids. In 3 of 13 remaining animals in the rhuApo2L/TRAIL group, microscopic findings consisted of minimal to mild individual cell necrosis in scattered hepatocytes. No macro- or microscopic observations were noted in rhuApo2L/TRAIL-treated animals at recovery necropsy.

ATAs were measured only by the RIP assay; as expected, no antibodies were detected in animals in the Vehicle group. While 2 of 14 animals in the rcynoApo2L/TRAIL group had detectable, but low-titer ATAs, ATAs were detected in 15 of 18 animals in the rhuApo2L/TRAIL group (Table 2). As observed in the previous study, clinical and/or microscopic evidence of hepatotoxicity was present only in ATA-positive animals. No indication of hepatotoxicity was noted in animals that were ATA-negative. Antibody titers were generally comparable in animals with or without hepatotoxicity. It was not possible to identify a threshold concentration of ATAs above which the likelihood of observing hepatotoxicity increased notably.

The crosslinking activity of the ATAs was again assessed in the Jurkat cytotoxicity assay. The anti-rcynoApo2L/TRAIL antibodies from the two ATA-positive animals in the rcynoApo2L/TRAIL group were unable to crosslink either rcynoApo2L/TRAIL or rhuApo2L/TRAIL (Figure 4d). In contrast, the antibodies against rhuApo2L/TRAIL were able to crosslink rhuApo2L/TRAIL and decrease Jurkat cell viability in 11 of 15 ATA-positive animals in the rhuApo2L/TRAIL group (Figure 4e and Table 2). Additionally, crosslinking activity was observed in 2 of 15 ATA-positive rhuApo2L/TRAIL-treated animals after incubation with rcynoApo2L/TRAIL (data not shown). As observed previously, the very high antibody titers in these two animals suggest some minimal nonspecific crossreactivity with rcynoApo2L/TRAIL. A correlation existed between the development of hepatotoxicity and the presence of ATAs that had cytotoxic crosslinking activity. The two animals in the rcynoApo2L/TRAIL group that were ATA-positive had no biochemical or microscopic evidence of toxicity and the ATAs exerted no crosslinking activity (as evidenced by the Jurkat cytotoxicity assay). In contrast, all five animals in the rhuApo2L/TRAIL group that developed hepatotoxicity had ATAs with strong crosslinking activity in the Jurkat cytotoxicity assay. However, not all ATA-positive animals developed hepatotoxicity.

## Discussion

Apo2L/TRAIL engages the extrinsic apoptosis pathway via binding to cell-surface death receptors. The sensitivity of tumor cells to Apo2L/TRAIL makes it attractive as a potential anticancer therapeutic, but drug development efforts have been hampered by literature reports of hepatotoxicity of non-optimized versions of Apo2L/TRAIL^23^ and by observations of hepatotoxicity as described in this report, as well as by an apparent lack of clinical efficacy.^18^

In the 4-week repeat-dose study, hepatotoxicity emerged as a subtle signal, impacting only 1 of 40 rhuApo2L/TRAIL-treated animals. In the affected animal, liver enzymes increased late in the study and corresponding microscopic evidence of hepatocellular damage was evident. These observations were interpreted at the time to be unrelated to rhuApo2L/TRAIL administration based primarily on their occurrence in a single animal.

The rhuApo2L/TRAIL clinical development plan included the possible treatment of patients with nephrotoxic chemotherapy agents. Thus, the safety of combined treatment with rhuApo2L/TRAIL was investigated using the prototypical nephrotoxicant, cisplatin. Although rhuApo2L/TRAIL did not exacerbate cisplatin-induced renal damage, the combination studies provided strong evidence of rhuApo2L/TRAIL-related hepatotoxicity in cynomolgus monkeys. Similar to the single affected animal from the 4-week study, progressive increases in liver enzymes were observed in a subset of animals. The magnitude of the changes increased with successive cycles of rhuApo2L/TRAIL dosing. Previous studies have shown an adverse effect of combined cisplatin+Apo2L/TRAIL treatment on hepatocytes *in vitro*.^27, 28, 29^ However, cisplatin administration did not appear to augment the effect of rhuApo2L/TRAIL treatment on the liver in our studies. Additionally, no evidence of a pharmacokinetic interaction between cisplatin and rhuApo2L/TRAIL was observed (data not shown); the mild renal injury induced by cisplatin treatment did not affect the clearance of rhuApo2L/TRAIL.

The incidence of rhuApo2L/TRAIL-induced hepatotoxicity rose with the increase in the number of treatment cycles. This, together with the observation that hepatotoxicity developed after multiple doses or cycles of rhuApo2L/TRAIL administration, is consistent with the features of a humoral immune response. Antibodies generally form within 10–14 days after initial antigen exposure (i.e., following the generation of plasma cells), and immune responses to foreign proteins are typically strengthened by repeated exposure to antigens (i.e., the ‘prime-boost' phenomenon). Human and cynomolgus monkey Apo2L/TRAIL differ by four amino acids (Figure 5a), three of which are clustered in an exposed region of the protein and could serve as an immunogenic epitope (Figure 5b). Thus, we hypothesized that rhuApo2L/TRAIL-induced hepatotoxicity is the result of the aggregation of hepatocyte death receptors by ATA-crosslinked rhuApo2L/TRAIL. By binding to multiple rhuApo2L/TRAIL ligands, anti-rhuApo2L/TRAIL antibodies can amplify downstream signaling leading to apoptosis in otherwise resistant hepatocytes (Figure 5c).^30^

Anti-rhuApo2L/TRAIL antibodies were assessed by ELISA in every study, but were largely undetected due to the low drug tolerance and poor sensitivity of this assay method. The RIP assay, which is approximately 25 times more sensitive than the ELISA and developed as an adjunct to the ELISA, was able to identify a greater number of ATA-positive animals. A cell-based bioassay, used to assess the activity of the RIP-detected antibodies, demonstrated that ATAs, in the presence of rhuApo2L/TRAIL, induced apoptosis in Jurkat cells which are normally resistant to rhuApo2L/TRAIL. Published reports of polyhistidine-tagged or leucine-zipper Apo2L/TRAIL (i.e., non-optimized versions of the ligand) decreasing the viability of the hepatocytes *in vitro* support the antibody crosslinking hypothesis of hepatotoxicity.^25^ These non-optimized versions are likely to aggregate death receptors at the hepatocyte cell surface similarly to ATA-crosslinked rhuApo2L/TRAIL, resulting in the amplification of the apoptotic signal. A recent report from a Phase I study of a novel DR5 receptor agonist, in which preexisting ATAs were associated with the development of hepatotoxicity (Grade ≥3 increases in ALT and/or AST) in several patients, supports this mechanism of toxicity. The investigators hypothesized that these events were related to immune complexes over-multimerizing receptors and amplifying the apoptotic signal on normally resistant hepatocytes.^31^ Likewise, Graves *et al.*^32^ described the formation of a ternary complex of Apo2L/TRAIL with the DR5 receptor and a DR5 agonist antibody. The combined administration of Apo2L/TRAIL and the antibody, via the formation of this complex and the resultant enhancement of apoptotic signaling, led to increased tumor cell killing *in vitro* and *in vivo*. These results further support the mechanism of toxicity proposed in the present report.

We tested the crosslinking ATA hypothesis by administering high-dose rhuApo2L/TRAIL or rcynoApo2L/TRAIL to cynomolgus monkeys for several cycles of treatment. As expected, rcynoApo2L/TRAIL was well tolerated. Antibodies to rcynoApo2L/TRAIL were detected in 2 of 14 animals, but their lack of *in vitro* crosslinking activity likely explains the observed *in vivo* tolerability of rcynoApo2L/TRAIL. In contrast, administration of rhuApo2L/TRAIL resulted in the elevation of liver enzymes with increased incidence and magnitude with successive cycles of treatment, and corresponding microscopic evidence of hepatotoxicity. Anti-rhuApo2L/TRAIL antibodies were detected in 15 of 18 animals, and crosslinking activity was confirmed in 11 of 15 ATA-positive animals. Of the five animals in the rhuApo2L/TRAIL group that developed clinical and microscopic evidence of hepatotoxicity, all had ATAs with strong crosslinking activity. However, as noted previously, not all animals with ATAs developed hepatotoxicity over the duration of treatment tested. Thus, the presence of ATAs against rhuApo2L/TRAIL appeared requisite to the development of hepatotoxicity.

Immunological responses to foreign proteins, including biotherapeutics, are complex. The antibody population is polyclonal and differences in epitope specificity as well as affinity and avidity exist. Although these factors were not the focus of the present studies, it is likely that they contribute to the observed incidence of hepatotoxicity in only a subset of ATA-positive animals. The results of these studies support the hypothesis that hepatotoxicity in monkeys administered rhuApo2L/TRAIL occurs as a result of the development of antibodies to a heterologous-species protein. Although the potential exists for patients to develop antibodies to rhuApo2L/TRAIL, it is unlikely that they would bind rhuApo2L/TRAIL via the same epitope as cynomolgus monkey antibodies because of the absence of the surface-exposed epitope in the autologous human setting. Correspondingly, human antibodies to rhuApo2L/TRAIL would be expected to have very little crosslinking activity and hepatotoxicity by this mechanism would not be expected in the clinical population. Importantly, single-nucleotide polymorphism analysis of >5000 sequences entered in the human genome databases revealed no protein sequence polymorphism in the ectodomain of human Apo2L/TRAIL. Thus, the administration of rhuApo2L/TRAIL to humans should not be immunogenic.

To date, drug-induced immune-mediated hepatotoxicity has typically been explained by the hapten hypothesis, which posits that metabolism produces a reactive intermediate that can bind to sulfhydryl groups in proteins. This modified protein (hapten) serves as an immunogenic stimulus.^33^ The present report details the observation of a distinct mechanism of immune-mediated hepatotoxicity occurring via the development of ATAs that facilitate a potent biological effect on hepatocytes. The successful evaluation of the hypothesis to explain rhuApo2L/TRAIL-associated hepatotoxicity enabled the filing of an IND with the US FDA and initiation of Phase I clinical trials. In light of the mechanism of toxicity observed in cynomolgus monkeys, real-time antibody monitoring was incorporated into the design of the Phase I study to ensure patient safety. Only 1 of 71 patients developed anti-rhuApo2L/TRAIL antibodies. No relationship between the presence of antibodies and adverse effects was noted. The safety of rhuApo2L/TRAIL in humans was demonstrated by a lack of clinically significant hepatotoxicity,^34, 35, 36^ further validating the nonclinical observations of species-specific immune-mediated hepatotoxicity and enabling the clinical evaluation of this oncology therapeutic.

## Materials and Methods

### Identification of amino acid sequence differences

Cynomolgus monkey Apo2L/TRAIL was cloned and sequenced at Genentech, Inc., and compared with the publicly available sequence of human Apo2L/TRAIL to identify critical amino acid differences in the extracellular domain (residues 114–281).

### Production of rhuApo2L/TRAIL

RhuApo2L/TRAIL consists of amino acids 114–281 of the extracellular domain of the natural ligand and includes a free cysteine at Position 230. X-ray crystallographic and other biophysical characterization data on the purified product indicate that rhuApo2L/TRAIL is a non-covalent, 60-kDa homotrimer, with a zinc ion coordinated to the three free cysteines and a chloride.^37^ RhuApo2L/TRAIL was expressed in *Escherichia coli* and grown in a non-serum-containing complex culture medium composed of casein hydrolysate, yeast extract, glucose, salts and trace elements. RhuApo2L/TRAIL accumulated in the cytoplasm and was released by the mechanical lysis of cells. The protein was then extracted from the whole-cell broth using homogenization and centrifugation and was further purified using several chromatographic steps. The purified protein was formulated by ultrafiltration and diafiltration. RhuApo2L/TRAIL was provided as a single-use lyophilized formulation containing 110 mg (105 mg to deliver) of rhuApo2L/TRAIL per 10 ml glass vial. Vials were reconstituted with 5 ml of sterile water for injection, resulting in a 20 mg/ml protein solution and a formulation composition of 0.5 M arginine succinate, 20 mM Tris, 0.02% polysorbate 20, pH 7.2.

### Production of cynomolgus monkey Apo2L/TRAIL

The recombinant cynomolgus monkey (rcyno) Apo2L/TRAIL molecule consists of amino acids 114–281 of the extracellular domain of the natural cynomolgus monkey sequence. Unlike rhuApo2L/TRAIL, rcynoApo2L/TRAIL was expressed as monomers that efficiently assembled into homotrimers involving zinc ion as a cofactor. RcynoApo2L/TRAIL was expressed in *E. coli*, processed via a method identical to that described for rhuApo2L/TRAIL, and formulated using the same excipient concentrations as rhuApo2L/TRAIL. The drug product was a liquid solution that was stored frozen.

### Animals

Experimentally naïve male and female cynomolgus monkeys of Chinese origin were obtained from Covance Research Products (Alice, TX, USA) or Scientific Resources International (Reno, NV, USA). Animals were 1.9–5.2 years of age at the outset of the study. Prior to the initiation of dosing, animals were acclimated to the laboratory conditions during at least a 30-day quarantine period. All animals selected for possible assignment to the study underwent a comprehensive physical examination prior to assignment. Animals were individually housed in stainless steel cages. Primary enclosures were as specified in the USDA Animal Welfare Act (9 CFR, Parts 1, 2 and 3) and as described in the Guide for the Care and Use of Laboratory Animals (Institute for Laboratory Animal Resources (ILAR) publication, 1996). Environmental controls were set to maintain a temperature range of 18–29 °C, relative humidity range of 30–70%, 10 or greater air changes/h and a 12 h light/12 h dark cycle. The light/dark cycle was interrupted as necessary for study related procedures. Certified primate diet (Harlan Teklad) was provided daily. Also, small amounts of fruit, cereal or other treats were occasionally given to the animals as part of the Testing Facility's environmental enrichment program. Tap water, via an automatic watering system, was available *ad libitum*. No contaminants were known to be present in the diet or water at levels that might have interfered with the study. Animals were assigned to groups by a stratified randomization scheme designed to achieve similar group mean body weights, and the groups were randomly assigned to the treatments. All animals were observed twice daily for changes in general appearance and behavior, mortality or morbidity. Individual animal body weights were measured once weekly. Individual animal food consumption was qualitatively assessed once daily. The timing of other assessments is discussed individually for each of the studies.

### Study design

All studies were conducted in compliance with international Good Laboratory Practice (GLP) standards. The principal goal of these studies was to evaluate the safety profile of rhuApo2L/TRAIL. The dosing regimens employed were chosen to support the proposed clinical use of rhuApo2L/TRAIL. In two studies, rhuApo2L/TRAIL was administered with the known nephrotoxicant, cisplatin, either concomitantly or sequentially, to test the potential effect of rhuApo2L/TRAIL in the setting of renal injury. The dose of cisplatin utilized was intentionally selected to induce mild renal damage to allow the detection of any potential rhuApo2L/TRAIL administration-related exacerbation of renal injury.

#### Four-week repeat-dose rhuApo2L/TRAIL study

Animals were administered rhuApo2L/TRAIL at dose levels of 0 (Vehicle), 10, 30 or 100 mg/kg by 1 h intravenous (IV) infusion either once daily for 28 days (*n*=4–6/sex/group) (daily-dosed group, Figure 1a; top line), or for five consecutive days per week every 3 weeks (5d q3wk) for two cycles (days 1–5 and 22–26; 100 mg/kg dose level only; *n*=6/sex/group) (intermittent 100 mg/kg group; Figure 1a, bottom line). Following overnight fasting, blood was collected on days 1, 7, 15 and 22 (for clinical chemistry and hematology/coagulation parameter analysis) and days 2 and 23 (for clinical chemistry analysis only) from the daily-dosed group animals. Physical examinations, including measurement of body temperature, heart and respiratory rates, and observations of the condition of integument, respiratory and cardiovascular systems, were conducted on all animals on day 1 at 1–3 h post-dose and during weeks 4 and 8 (just prior to necropsy). For ATA assessment, blood was collected prior to dosing on days 1 (all groups), 22 and 27 (intermittent 100 mg/kg group), 29 (all daily-dosed groups), 42 (all groups), 54 (intermittent 100 mg/kg group) and 56 (all daily-dosed groups). Terminal necropsies were conducted one day after the completion of dosing (day 29 for daily-dosed groups and day 27 for the intermittent 100 mg/kg group). Recovery necropsies (*n*=2/sex/group for the control, daily high dose and intermittent high-dose groups) were conducted following the 4-week treatment-free period (day 56 for daily-dosed group and day 54 for the intermittent 100 mg/kg group, respectively).

#### Concomitant cisplatin+rhuApo2L/TRAIL administration

Animals were treated concomitantly with cisplatin+rhuApo2L/TRAIL in two separate studies. In the first study (Figure 2a, top line), animals were administered cisplatin (4 mg/kg) by 15 min IV infusion on days 1 and 22. Immediately after the cisplatin dose, the catheter was flushed with 2 ml of sterile saline. Animals then received rhuApo2L/TRAIL at dose levels of 0 (Vehicle), 30 or 100 mg/kg by 1 h IV infusion (*n*=2/sex/group) on days 1–5 and 22–26. Following overnight fasting, blood for serum chemistry, hematology and coagulation parameter analysis was collected prestudy and on day 27. Blood for serum chemistry analysis was collected on days 2, 6, 22 and 23; blood for serum chemistry and hematology analysis was collected on day 15. Physical examinations including observations of the condition of integument, respiratory and cardiovascular systems (including 6-lead electrocardiogram) were conducted on all animals 1 h post-dose on days 1 and 26. Blood was collected for ATA analysis prestudy, predose on days 22 and on day 27. Necropsies were conducted on day 27.

In the second study (Figure 2a, bottom line), animals (*n*=6/sex) were administered cisplatin (4 mg/kg) by 15 min IV infusion on days 43 and 64. Immediately after the cisplatin dose, the catheter was flushed with 2 ml of sterile saline. Animals then received rhuApo2L/TRAIL (100 mg/kg) by 1 h IV infusion on days 43–47 and 64–68. Following overnight fasting, blood for serum chemistry, hematology and coagulation parameter analysis was collected prestudy and on day 63. Blood for evaluation of serum chemistry and hematology was collected on days 42 and 84. Blood for evaluation of serum chemistry only was collected on days 2, 12, 21, 23, 44, 53, 65, 74 and 86. Physical examinations including observations of the condition of integument, respiratory and cardiovascular systems were conducted on all available animals on day 85 or 86. Blood was collected for ATA analysis prestudy and on day 84. Owing to toxicity, necropsies were conducted on all animals on day 85 or 86.

#### Sequential cisplatin+rhuApo2L/TRAIL administration

Animals were administered cisplatin (4 mg/kg) by 15 min IV infusion on days 1 and 22 (Figure 3a). Immediately after the cisplatin dose, the catheter was flushed with 2 ml of sterile saline. RhuApo2L/TRAIL was administered by 1 h IV infusion at doses of 0 (Vehicle), 10, 30 or 100 mg/kg (*n*=4–6/sex/group) on days 43–47, 64–68, 85–89 and 106–110.

Following overnight fasting, blood for serum chemistry, hematology and coagulation parameter analysis was collected prestudy and on days 63, 111 and 127. Blood for evaluation of serum chemistry and hematology was collected on days 42, 84 and 105. Blood for evaluation of serum chemistry only was collected on days 2, 12, 21, 23, 44, 53, 65, 74, 86, 89, 95, 107 and 110. Physical examinations, including observations of the condition of integument, respiratory and cardiovascular systems, were conducted on all the available animals on day 111 or 127. Blood was collected for ATA analysis prestudy and on days 84, 105, 111 and 127. Terminal necropsies were conducted one day after the completion of dosing (day 111) in the sequential treatment group. Recovery necropsies (*n*=1–2/sex/group for animals in the control and high-dose treatment group) were conducted following a 16-day treatment-free period (day 127).

#### Investigative (rhuApo2L/TRAIL *versus* rcynoApo2L/TRAIL) toxicology study

Animals were administered Vehicle, rhuApo2L/TRAIL or rcynoApo2L/TRAIL (100 mg/kg) (*n*=4–9/sex/group) on days 1–5, 22–26, 43–47 and 64–68 (Figure 4a). Following overnight fasting, blood for clinical chemistry, hematology and coagulation parameter analysis was collected prestudy and on days 42, 69 and 85. Blood for clinical chemistry analysis was additionally collected on days 2, 8, 15, 21, 23, 29, 36, 44, 50, 57, 63, 65 and 77. Physical examinations including observations of the condition of integument, respiratory and cardiovascular systems were conducted prestudy and just prior to euthanasia on days 69 or 85. Blood was collected for ATA analysis prestudy and on days 8, 21, 29, 42, 50, 63, 69, 71 and 85. Terminal necropsies were conducted one day after the completion of dosing (day 69). Recovery necropsies (*n*=1–2/sex/group in the control and rhuApo2L/TRAIL groups) were conducted following a 16-day treatment-free period (day 85).

### Antitherapeutic (rhuApo2L/TRAIL) antibody assay

#### Enzyme-linked immunosorbent assay (ELISA)

Microtiter plates (NUNC Maxi-Sorp, Rochester, NY, USA) were coated with a rabbit anti-rhuApo2L/TRAIL polyclonal antibody, and then incubated at 2–8 °C from 12 to 72 h. After the plates were blocked, standards, serum samples and controls were added to the plate and incubated for 1–2 h. Captured rhuApo2L/TRAIL was detected with a biotinylated monoclonal antibody (Mab 5C2). Next, strepavidin horseradish peroxidase (Southern Biotech, Birmingham, AL, USA) was added. For color development, tetramethyl benzidine peroxidase substrate (two-step TMB; KPL, Inc., Gaithersburg, MD, USA) was added. Lastly, 1 M phosphoric acid was added to stop the reaction and plates were read at an absorbance of 450 nm (Molecular Devices Emax plate reader, Sunnyvale, CA, USA). The minimum quantifiable concentration in this assay was 62.5 ng/ml. Accuracy, intra-assay precision and inter-assay precision were acceptable during the validation experiments.

#### RIP assay

Serum samples at a minimum dilution of 1 : 50 were added to an equal volume of ^125^I-labeled rhuApo2L/TRAIL or rcynoApo2L/TRAIL, resulting in a 1 : 100 final minimum dilution of the serum. After 15–18 h incubation at 2–8 °C, goat anti-human Ig antiserum was added to each sample and incubated for 2 h at ambient temperature to precipitate the human Ig/goat anti-human Ig immune complexes. Any ^125^I-rhuApo2L/TRAIL-anti-rhuApo2L/TRAIL antibody complexes present in the serum sample were pelleted with human Ig/goat anti-human Ig immune complexes by centrifugation at 2000 *× g* for 20 min at 4 °C. The supernatant was decanted, and the radioactivity associated with the pellet was measured using a gamma counter (Perkin Elmer Packard Cobra II, Waltham, MA, USA). The percent ^125^I bound compared to the total input ^125^I (%B/T) was determined for each sample dilution in the assay. The assay cut point was determined as two times the %B/T for the negative control sample (1% normal human sera in sample diluent) in the assay. The titer for an antibody positive sample was determined as the log_10_ of the sample dilution that results in the %B/T equal to the assay cut point. Because the minimum dilution of each sample was 1 : 100, the minimum detectable antibody titer was 2 (log_10_ of 100).

### Jurkat T-cell cytotoxicity assay

Jurkat T leukemia cells were maintained in RPMI 1640 medium (Invitrogen, Carlsbad, CA, USA) supplemented with 10% fetal bovine serum (Invitrogen, Carlsbad, CA, USA). Cynomolgus monkey serum was incubated with rhuApo2L/TRAIL (Genentech, Lot 35992-14) or rcynoApo2L/TRAIL (Genentech, Lot P Cyno Apo2L #1) for 30 min at 37 °C at dilutions ranging from 1 : 100 to 1 : 819 200. After this incubation, Jurkat T cells were added to the wells and incubated for 24 h at 37 °C. As a positive control, Jurkat cells were incubated for 24 h at 37 °C with human (Genentech, Lot 35922-44) or cynomolgus (Genentech, Lot 35992-89B) Flag-tagged Apo2L/TRAIL crosslinked with M2 (Sigma, St Louis, MO, USA), a monoclonal antibody against the Flag tag. The fluorometric/colorimetric growth indicator Alamar Blue (Trek Diagnostic Systems, Inc., Cleveland, OH, USA) was added to the wells for the last 4–6 h of the incubation period. Alamar Blue is a blue, nonfluorescent redox dye in its oxidized state. When taken up by the living cells, intracellular metabolic reduction converts it to a fluorescent red color. The fluorescence is proportional to the metabolic activity and, indirectly, to the number of viable cells. Fluorescence was read using a 96-well fluorometer (Molecular Devices SPECTRAmax M2, Sunnyvale, CA, USA) with an excitation at 530 nm and emission of 590 nm. The minimum sample dilution was 1 : 100. Thus, the sensitivity of the assays was log_10_ of the 100-fold dilution (2.0 titer units). Titer values were calculated and reported as the reciprocal of log10 of the sample dilution that reduced Jurkat cell viability by 50%. Titer for samples that produced no reduction or a reduction in viability of less than 50% was reported as <2.

## Figures and Tables

**Table 1:** Correlation of anti-rhuApo2L/TRAIL antibodies and hepatotoxicity in cynomolgus monkey toxicology studies

**Study**	**rhuApo2L/TRAIL dose level (mg/kg)**	**No/Group**	**No. with ATA titer – ELISA**	**No. with ATA titer – RIP**	**No. with crosslinking activity**^a^	**No. with ALT increase**^b^	**No. with liver histopathology**	**No. of treatment-related deaths**^c^
4-week repeat-dose study	0	12	0	NT	NT	0	0	0
	10	8	0	NT	NT	0	0	0
	30	8	0	NT	NT	0	0	0
	100^d^	12	0	NT	NT	1	1	0
	100^e^	12	0	NT	NT	0	0	0
Concomitant renal injury model	0	4	0	NT	NT	0	0	0
	30	4	0	NT	NT	1	2	0
	100	4	0	NT	NT	2	1	1
	100^f^	12	3	7	6	11	2	2
Sequential renal injury model	0	9	0	0	0	0	0	0
	10	8	1	1	1	1	0	0
	30	8	1	6	2	2	2	2
	100	12	5	10	5	4	3	1^g^

Abbreviation: NT, not tested.

a Crosslinking activity determined by Jurkat cytotoxicity assay.

b Increase in ALT greater than twice the upper limit of the normal range.

c Deaths related to rhuApo2L/TRAIL hepatotoxicity.

d Daily administration for 28 days.

e Two cycles of treatment (5d q3wk) over 28 days.

f Concomitant cisplatin+100 mg/kg rhuApo2L/TRAIL group from second renal injury model study.

g Animal died of a study procedure-related hemorrhage; severe hepatocellular necrosis was observed microscopically but was not the cause of death *per se*.

**Table 2:** Correlation of anti-rhuApo2L/TRAIL antibodies and hepatotoxicity in rcynoApo2L/TRAIL *versus* rhuApo2L/TRAIL mechanism of toxicity study

**Dose group**	**No./group**	**No. with ATA titer**^a^	**No. with crosslinking activity**^b^	**No. with ALT increase**^c^	**No. with liver histopathology**	**No. of treatment-related deaths**
Apo2L/TRAIL Vehicle	8	0	0	0	0	0
rcynoApo2L/TRAIL	14	2	0	0	0	0
rhuApo2L/TRAIL	18	15	11	9	5	1

a ATA titer determined by radioimmunoprecipitation assay.

b Crosslinking activity determined by Jurkat cytotoxicity assay.

c Increase in ALT greater than twice the upper limit of the normal range.

## Abbreviations

ALP: alkaline phosphatase
ALT: alanine aminotransferase
AST: aspartate aminotransferase
ATA: antitherapeutic antibody
CP: clinical pathology
DR: death receptor
ELISA: enzyme-linked immunosorbent assay
H&E: hematoxylin and eosin
Ig: immunoglobulin
ILAR: Institute for Laboratory Animal Resources
IND: investigational new drug application
IV: intravenous or intravenously
*n*: number
N: necropsy
NT: not tested
OPG: osteoprotegerin
q3w: every 3 weeks
rcyno: recombinant cynomolgus monkey
rhu: recombinant human
RIP: radioimmunoprecipitation
RN: recovery necropsy
TN: terminal necropsy
TNF: tumor necrosis factor
ULN: upper limit of normal
USDA: United States Department of Agriculture
US FDA: United States Food and Drug Administration
